# Mechanistic insights into substrate-targeting modification effects on interfacial kinetics of raw starch-degrading amylases

**DOI:** 10.1128/aem.01908-25

**Published:** 2026-04-06

**Authors:** Lingli Zhong, Zongchao Huo, Min Jiang, Wenming Chang, Kai Yang, Xue Chen, Xianfeng Ye, Yanling Ji, Yan Huang, Lei Zhang, Zhoukun Li, Yanwei Li, Zhongli Cui

**Affiliations:** 1 Key Laboratory of Agricultural Environmental Microbiology, Ministry of Agriculture and Rural Affairs, College of Life Sciences, Nanjing Agricultural University 70578, Nanjing, China; 2 Environment Research Institute, Shandong University 547481, Qingdao, China; Shanghai Jiao Tong University, Shanghai, China

**Keywords:** raw starch-degrading enzymes, starch granules, substrate-targeting specificity, interfacial catalysis kinetics, amylopectin double helices

## Abstract

Current research on interfacial enzyme catalysis of starch granules largely overlooks interactions with molecules of specific spatial conformations. Here, we demonstrated that the CBM20-truncated mutant AmyM-TR2 exhibited over 30-fold greater hydrolysis efficiency toward starch granules compared to wild-type AmyM, with a shifted preference toward amylopectin. Molecular docking revealed that the exposed domain C in AmyM-TR2 enables binding to double-helical α-glucan molecules by three key residues (R351, W374, and W392). Interfacial catalytic analysis revealed that the discrepancy of AmyM and AmyM-TR2 in hydrolysis efficiency toward starch granules was related to substrate-targeting specificity and corresponding targeted productive binding sites (PBSs). AmyM-TR2 demonstrated a superior sustained catalytic capacity toward its targeted PBSs within multi-component starch. The processive catalytic capacity of AmyM-TR2 toward amylopectin double helices in starch granules is the fundamental determinant of its highly efficient degradation, providing new insights for interfacial catalytic mechanisms and modification of interface enzymes.

**IMPORTANCE:**

Interfacial enzyme reactions are ubiquitous in both natural processes and industrial applications. Understanding the mechanistic aspects of enzymatic action on insoluble substrates is crucial for advancing sustainable biomass conversion and waste treatment technologies. Starch granule degradation mediated by raw starch-digesting enzymes (RSDEs) is a classical interfacial enzymatic reaction. The mechanism of RSDEs on starch granules is attractive, as it effectively circumvents the high energy consumption and viscosity challenges associated with conventional high-concentration starch hydrolysis processes. In this study, we demonstrate interfacial catalysis in starch granule depolymerization by substrate-targeting modification in raw starch-degrading amylase AmyM by exposing novel starch-binding modules, thus establishing a critical link between catalytic characteristics of amylase and starch substrate properties, which provides new insight for the contributions to insoluble substrate degradation within heterogeneous systems.

## INTRODUCTION

Raw starch-digesting enzymes (RSDEs) enable direct hydrolysis of native starch granules below gelatinization temperatures, thereby reducing energy consumption and production costs in industrial applications (1). However, only ⁓10% of amylolytic enzymes exhibit the capacity to function as RSDEs, requiring the presence of starch-binding domains (SBDs) and/or surface binding sites (SBSs) (2). Currently, 15 carbohydrate-binding domain (CBM) families possess the SBD function in the CAZy database: CBM20, 21, 25, 26, 34, 41, 45, 48, 53, 58, 68, 69, 74, 82, and 83 (3). Certain CBM-lacking RSDEs achieve raw starch hydrolysis through SBSs, leveraging aromatic residue-mediated stacking interactions during substrate binding. Although SBDs or SBSs can promote the decomposition of starch granules, the mechanisms that account for their functions are only partially understood.

As one of the most abundant natural polysaccharides, starch exhibits more complex molecular structures and hierarchical organizations than cellulose, mainly composed of amylose and amylopectin. The adjacent chains of highly branched amylopectin molecules associate to form double helices to pack into crystalline regions, which enable starch to exist as semicrystalline insoluble granules (4). RSDE-mediated raw starch degradation represents a classical interfacial enzyme reaction, occurring at the interface between starch granules and water. However, interfacial enzyme reactions challenge these classical kinetic models due to dynamic substrate concentrations and uncertain quasi-steady-state assumption under heterogeneous systems. Recent advances propose modified models and concepts of interfacial enzyme kinetics, primarily validated in cellulase–cellulose systems (5–7). Meanwhile, only sporadic studies have employed improved Michaelis–Menten kinetics to probe enzymatic interactions at the RSDE–starch granule interface (8, 9). The interfacial enzyme reactions between RSDEs and starch granules are more complicated than cellulase–cellulose systems. Hence, elucidating the mechanisms between RSDEs and starch granules holds both theoretical and practical significance for advancing interfacial catalysis technologies and industrial applications.

In our previous studies, amylase AmyM derived from *Corallococcus* sp. EGB was identified as an efficient RSDE, demonstrating approximately 95% conversion efficiency toward 30% (wt/vol) raw starch (10). In the subsequent application evaluation, the CBM20-truncated mutant AmyM-TR2 exhibited stronger disruption effects on dough structures than wild-type AmyM (11). In this study, we found that the CBM20-truncated mutant AmyM-TR2 shifted the enzymatic hydrolysis preference from amylose to amylopectin, resulting in a disproportionate enhancement (over 30-fold) in starch granule hydrolysis efficiency. The specific binding between the exposed domain C and amylopectin double helices was investigated and validated through molecular docking. To investigate the interfacial catalytic mechanism of AmyM-TR2, we compared the binding and catalytic kinetic properties between AmyM-TR2 and AmyM using various starch substrates.

## MATERIALS AND METHODS

### Materials

The host strains *Escherichia coli* DH5α and *Pichia pastoris* GS115 and vector pEFαA were procured and used for cloning and protein expression. The maltohexaose-forming α-amylase AmyM from *Corallococcus* sp. EGB and its truncation AmyM-TR2 were prepared in *P. pastoris* GS115 as described before (10). All chemicals used in this study were of analytical grade purchased from Sinopharm Chemical Reagent Co., Ltd. (Shanghai, China).

Soluble starch (PSS) was purchased from Solarbio Science & Technology Co. Ltd. (Beijing, China). Normal wheat starch (NWS), normal corn starch (NCS), and high-amylose starch extracted from corn (CHAM) were generously provided by Yuanye Biotechnology Co., Ltd. (Shanghai, China). Amylopectin (CAP) and amylose (CAM) were obtained from TCI (Shanghai) Development Co., Ltd., and Shanghai Titan Scientific Co., Ltd. (Shanghai, China), respectively. According to previous studies, PSS and NWS were identified as the preferred substrates under gelatinized and granular conditions, respectively (10, 12). In the granular-starch system, all substrates remained insoluble in water; no free sugars were detectable in the supernatant (within the detection limit of the DNS assay). The apparent amylose content (AAC) of starch granules was previously determined by iodine colorimetry (Table 1).

**TABLE 1:** Characteristics of starch granules

Type of starch	Source	Abbreviation	AAC (%)
Soluble starch	Potato	PSS	6.0
Normal wheat starch	Wheat	NWS	24.3
Normal corn starch	Corn	NCS	38.5
Amylopectin	Waxy corn	CAP	<1
Amylose	Corn	CAM	98.0
High-amylose starch	Corn	CHAM	73.7

### Construction, expression, and purification of mutants

Based on the plasmid pEFαA-*amyM-TR2*, recombinant plasmids (the R351A, W374A, P376A, W380A, and W392A mutations) were generated by using the Mut Express II Fast Mutagenesis Kit version 2 (Vazyme, China) with primers listed in Table S1 (11). The recombinant plasmids were linearized with *Sca* I and transformed into *P. pastoris* GS115. The transformants were screened and isolated on YPD plates containing 100 μg/mL zeocin. The calculated molecular weights of AmyM and AmyM-TR2 were 53.2 and 42.8 kDa, respectively (https://web.expasy.org/). Protein quantification was performed using the Bradford method, with bovine serum albumin (BSA) serving as the standard protein for calibration . The purity of AmyM, AmyM-TR2, and the mutants of AmyM-TR2 were verified by SDS-PAGE (Fig. S1). Due to the removal of CBM20 containing the main glycosylation site, purified AmyM-TR2 migrated as a single band with a slightly higher apparent molecular weight (45 kDa) than predicted (Fig. S1A).

### Activity assays

To determine the standard activity toward gelatinized starch, the reaction mixture containing 5 mg/mL PSS and the purified enzyme was incubated at 50°C for 10 min in 20 mM pH 7.0 Tris-HCl buffer (12). Starch substrates were pre-gelatinized to form a well-dispersed sol with good fluidity under the assay conditions. The standard activity toward starch granules was determined with slight modifications to a previously described method (10). The reaction mixture containing 20 mg/mL NWS and purified enzyme was incubated at 50°C with shaking at 200 rpm for 15 min in 20 mM pH 7.0 Tris-HCl buffer.

The amount of reducing sugar was measured using the DNS method to determine the enzymatic activity of the recombinant amylase, as previously described (13). Glucose was used as a standard to quantify the amount of released sugar. The reaction was stopped by adding the DNS reagent (1:1, vol/vol) and incubated in boiling water for 5 min. Following cooling to room temperature, absorbance measurements were recorded at 540 nm using a microplate reader (SpectraMax i3x; Molecular Devices, California, USA). One unit of α-amylase activity was defined as the amount of enzyme required to liberate 1 μmol of reducing sugar per second.

### Sequence analysis and homology modeling

The phylogenetic analysis was performed using the neighbor-joining method in MEGA11. Branch confidence values were estimated using 1,000 bootstrap replicates of the original sequence data. Functional conserved domains were identified through the Conserved Domain Database (https://www.ncbi.nlm.nih.gov/cdd/). The resulting phylogenetic tree incorporating domain architectures was visualized using the iTOL platform (http://itol.embl.de/). The evolutionary classification of amylases was analyzed by referring to the CAZy database (http://www.cazy.org/).

The three-dimensional structural models of AmyM and its mutants were constructed using AlphaFold3 and subsequently validated with PROCHECK and ERRAT (14). To investigate the conserved amino acid motifs, sequence alignment of the GH13_6 subfamily was conducted using Clustal W and rendered by ESPript 3.0 (https://espript.ibcp.fr) (15). The aligned conserved motifs were then visualized through WebLogo 3.0 (http://weblogo.threeplusone.com/) (16).

### Biochemical properties of amylase

The optimal pH of purified amylase was determined under standard assay conditions using 20 mM buffer systems: citrate (pH 3.0–6.0), phosphate-buffered saline (pH 6.0–8.0), Tris-HCl (pH 7.0–9.0), and glycine-NaOH (pH 9.0–10.0). For pH stability assessment, the enzyme was incubated in respective buffers at 4℃ for 12 h before residual activity measurement. The optimal temperature was evaluated across a 30°C–80°C range under standard assay conditions. Thermostability was analyzed by monitoring residual activity after pre-incubating the enzyme at different temperatures (30°C–80°C) for 90 min. Untreated enzyme activity served as the control (100%).

The substrate specificity of purified amylase toward gelatinized starch was analyzed at the optimal pH and temperature conditions for 10 min using different substrates (PSS, NCS, CAP, CAM, and CHAM). To assess enzyme activity on starch granules, the substrate specificity was examined under optimal conditions for 15 min using NWS, NCS, CAP, CAM, and CHAM as substrates.

According to previously reported methods, the hydrolysis products of gelatinized starch (PSS) and starch granules (NWS) were analyzed using thin-layer chromatography and high-performance liquid chromatography (17). The enzymatic hydrolysis was performed under optimal conditions using amylase at a dosage of 0.1 U/mg starch for 24 h. The mixture of malto-oligosaccharides from glucose (G1) to maltoheptaose (G7) was used as the standard.

### Hydrolysis efficiency toward starch granules

The hydrolysis efficiency of AmyM and AmyM-TR2 toward 10% (wt/vol) corn starch was evaluated by measuring the residual starch granule mass (*W*_1_, dried to constant weight) at various time intervals. With slight modifications to the method reported in previous studies, the reaction mixture, consisting of 0.2 U/mg starch granules (enzyme dosage) and 2 mM CaCl_2_ in 20 mM Tris-HCl buffer (pH 6.0), was maintained at 45°C (10). Under the above conditions, the conversion efficiency of AmyM and AmyM-TR2 toward 10% NWS and NCS was evaluated across enzyme dosages ranging from 0.025 to 0.4 U/mg starch granules. The initial mass of starch granules was designated as *W*_0_. The degree of hydrolysis (DH, %) was defined as the ratio of hydrolyzed starch mass to the initial starch mass during the hydrolysis process, calculated using equation 1:


(1) $$\text{DH}\;(\%)=\frac{W_{0}-W_{1}}{W_{0}}\times 100$$

### Adsorption of enzymes toward starch granules

The binding position of enzymes on starch granules was performed by confocal laser scanning microscopy (CLSM) (18, 19). The enzyme (0.05 mM) reacted with 0.5 mM iFluor 488 succinimidyl ester (AAT Bioquest, USA) in 100 mM phosphate-buffered saline (pH 9.0), followed by incubation in the dark at room temperature for 1 h. The iFluor 488-labeled enzyme was purified using a Sephadex G-25 column (Smart-Lifesciences, China). The specific activity of the labeling enzyme was unchanged. Starch granules (10 mg/mL NCS) were mixed with 250 nM iFluor 488-labeled enzyme and incubated at 16°C for 30 min. After centrifugation (5,000 rpm, 30 s), the fluorescence detection of starch samples was investigated by CLSM (Leica TCSSP3, Germany) with an excitation wavelength of 488 nm and an emission wavelength of 507 nm. The experimental groups were compared against two control groups: untreated normal corn starch granules and starch granules suspension incubated with iFluor 488-labeled BSA.

The starch component for preferential hydrolysis on the granule surface was investigated by amylose content determination and Raman spectroscopy. The amylose content was measured by iodine colorimetry using the amylose content assay kit (BC4260; Beijing Solarbio Science & Technology Co., Ltd.). Raman spectra were recorded for enzyme-treated corn starch samples (DH value about 15%) using a confocal Raman microscope (Alpha300R; WITec GmbH, Germany) equipped with a TEM single-frequency laser (WITec GmbH). The excitation source was a 632.8 nm He-Ne laser with a power of 20 mW. The laser light was focused through a 50× oil immersion objective (Carl Zeiss, Germany) onto samples with the 600 g/mm grating. Raman mapping was acquired with 1 s integration time and 0.5 μm step size. All spectra were subjected to baseline correction, and the band area was normalized by the glucose ring signal at 476 cm^−1^ for comparison. Data analysis was conducted using Project 5 software (WITec GmbH) and OMNIC 8.0 software (Thermo Fisher Scientific, USA).

Based on previously reported methods, a Langmuir adsorption isotherm analysis was performed to evaluate the binding of the enzyme to starch granules (9, 18). To prevent starch granule modification and non-specific adsorption, the enzyme binding capacity of various starch granules (NCS, CAP, CAM, and CHAM) was determined at 16℃ containing 0.1 mg/mL acarbose and 0.2 mg/mL BSA. Enzyme concentrations were varied in the range of 0.2–7 μM while maintaining 25 mg/mL starch granules in 20 mM Tris-HCl (pH 7.0) with constant shaking at 100 rpm. After 30 min of incubation, the mixtures were centrifuged (12,000 rpm, 5 min), and the concentration of non-bound enzyme, E_free_, in the supernatant was determined by the Bradford method. Data were fitted to Langmuir adsorption isotherm using equation 2, where *Γ* was measured as (*E*_0_ − *E*_free_)/^mass^*S*_0_; *K*_d_ is the dissociation constant; and ^ads^*Γ*_max_ is the (apparent) saturation coverage (9):


(2) $$\Gamma =\frac{{}^{abs}\Gamma_{max}\times E_{free}}{K_{d}+E_{free}}$$

### Kinetic analysis of gelatinized starch and granular starch

For the enzymatic kinetics study on gelatinized starch, the conventional Michaelis–Menten (CMM) parameters were determined using varying substrate concentrations (PSS, NCS, CAP, CAM, and CHAM; 1–20 mg/mL). The conventional kinetic analyses were performed at 50°C in 20 mM Tris-HCl buffer (pH 7.0) containing 0.4 nM enzyme. The produced reducing sugar and added enzyme were quantified as described above. Initial reaction rates were determined from linear progress curves of experimental data, followed by non-linear regression analysis against equation 3 using Origin software 8.0 (Origin Lab, USA). This analysis returned values of *K*_m_ (in mg/mL) and *k*_cat_ (in s^−1^):


(3) $$\nu_{0}=\frac{k_{cat}\times E_{0}\times S_{0}}{K_{m}+S_{0}}$$

The interfacial kinetics on insoluble substrates were investigated using two complementary analytical approaches: CMM and inverse Michaelis-Menten (IMM) kinetics (5). In CMM experiments, the starch granule suspensions (NCS, CAP, CAM, and CHAM) were tested at concentrations ranging from 10 to 300 mg/mL. Reactions were conducted at 50°C with constant shaking at 200 rpm, maintaining a fixed enzyme concentration of 12 nM. The substrate hydrolysis degree was controlled below 1% during measurements to ensure initial rate conditions. For IMM kinetic analysis, enzyme concentrations were systematically varied between 4 and 125 nM while maintaining a constant substrate concentration of 25 mg/mL starch granule suspension under identical reaction conditions.

Experiments with substrate mass concentration as the independent variable were analyzed using the CMM equation:


(4) $$\nu_{0}=\frac{{}^{conv}k_{cat}\times E_{0}\times {}^{mass}S_{0}}{K_{1/2}+{}^{mass}S_{0}}$$

where ^mass^*S*_0_ is the substrate mass load and *K*_1/2_ is the mass load at substrate half-saturation. Using equation 4 for non-linear regression analyses of data, we found that this analysis returned values of *K*_1/2_ (in mg/mL) and ^conv^*k*_cat_ (in s^−1^):


(5) $$\nu_{0}=\frac{{}^{inv}V_{max}\times E_{0}}{K_{M}+E_{0}}$$

To analyze the IMM experiments, equation 5 was used in the non-linear regression analysis of data, giving the parameters *K*_M_ (in nM) and ^inv^*V*_max_ (in nM/s). *K*_M_ is the molar concentration of attack sites that gives half saturation of the enzyme, and ^inv^*V*_max_ is the maximum reaction rate at the attack site saturation. The reaction rate at the attack site saturation will be the product of the two parameters, as shown in equation 6:


(6) $${}^{inv}V_{\max}={\;}^{inv}k_{cat}\times {\;}^{mass}S_{0}$$

The parameter ^kin^*Γ*_max_ (in nmol/g) was introduced to quantify the number of attack sites for the enzyme per gram of substrate. By utilizing the experimentally determined values of *K*_1/2_ (from CMM) and *K*_M_ (from IMM), the attack site density (^kin^*Γ*_max_) can be determined by equation 7:


(7) $$K_{M}=K_{1/2}\times^{kin}\Gamma_{max}$$

### Molecular docking

The double-helical α-glucan molecule composed of maltodecaose (G12) subunits was extracted from the co-crystal structure (PDB ID: 7UWV). The ligand was preprocessed with AutoDock Tools to assign atomic charges, optimize rotatable bonds, and generate necessary input files. Molecular docking simulations between the double-helical α-glucan molecule and the enzyme mutants were executed via AutoDock Vina, with a grid box (30 × 30 × 30 Å) centered on catalytic residues homologous to the Sas6 protein (20). The optimal docking result was selected based on program scoring. Post-docking analysis was executed in Visual Molecular Dynamics to visualize binding poses, hydrogen bonding networks, and steric interactions.

### Statistical analysis

Experimental data were expressed as mean ± standard deviation from triplicate determinations. Statistical analyses were performed using SPSS version 22 (SPSS Inc., Chicago, IL, USA). All data were analyzed using Duncan’s multiple range test with statistical significance set at *P* < 0.05.

## RESULTS

### Removal of CBM20 in AmyM promotes starch granule hydrolysis

By comparing the hydrolysis properties toward gelatinized substrate (5 mg/mL PSS) and granular substrate (20 mg/mL NWS), AmyM and the truncated mutant AmyM-TR2 exhibited similar optimal hydrolysis conditions (50°C, Tris-HCl buffer, pH 7.0–8.0) and hydrolysis product patterns (Fig. S2 and S3). The presence of CBMs in amylase always benefits the thermal stability of its catalytic domain (21); thus, CBM20 truncation in AmyM resulted in decreased thermal stability. Compared to the wild type, AmyM-TR2 exhibited poorer thermal stability at temperatures above 50°C (Fig. S2E). These results indicated that CBM20 truncation did not alter the enzymatic hydrolysis properties but significantly reduced its thermal stability.

Removal of the SBD typically compromises granular starch hydrolysis more severely than gelatinized starch degradation (22). Contrary to current reports, AmyM-TR2 unexpectedly displayed higher activity than AmyM against all granular substrates, alongside a 14%–52% reduction in enzymatic activity toward gelatinized substrates (Table S2). The activity of AmyM-TR2 toward granular CHAM increased by 8.66-fold, along with approximately 2-fold improvements for NWS, NCS, CAP, and CAM.

**Fig 1:**
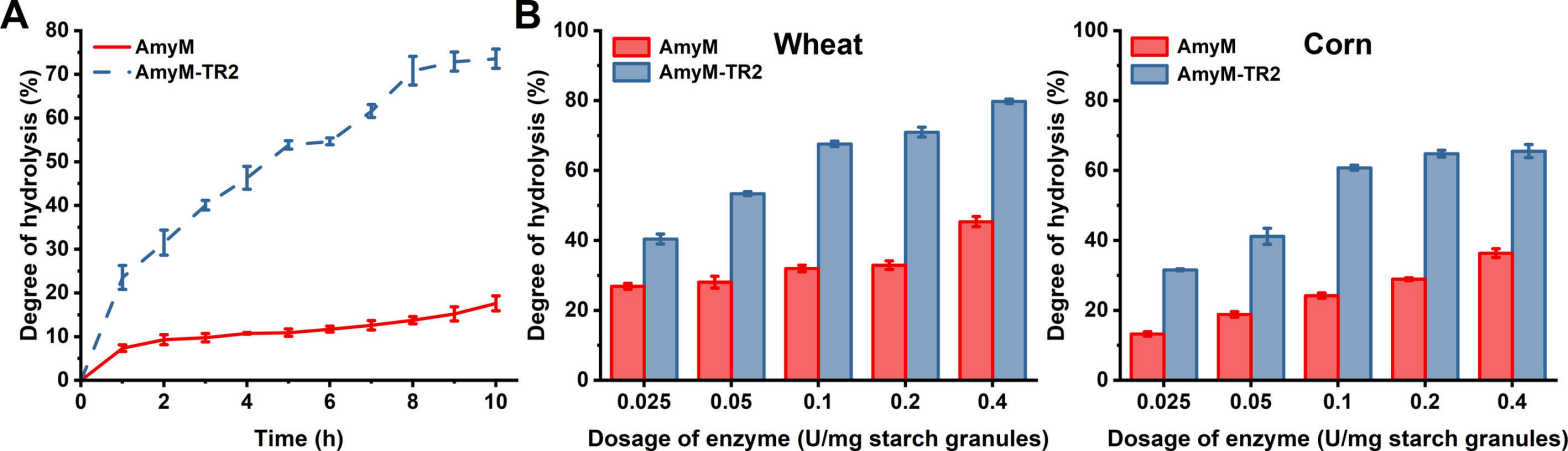
Starch granule hydrolysis of AmyM and the truncated mutant AmyM-TR2. (**A**) Hydrolysis curve of AmyM and AmyM-TR2 toward 10% normal corn starch with an enzyme dosage of 0.2 U/mg starch. (**B**) Effects of enzyme dosage on the degree of hydrolysis of AmyM and AmyM-TR2 toward 10% normal wheat starch and normal corn starch for 12 h. The starch granule hydrolysis reaction system was performed at 45°C and pH 6 with 2 mM CaCl_2_.

High enzymatic activity toward starch granules does not always guarantee high hydrolysis efficiency. As the currently reported RSDE with the highest activity, AmyZ1 yielded less than 50% starch granule conversion after 24 h, even when the enzyme dosage was increased to 10 U/mg starch (1). To evaluate the impact of CBM20 removal on starch granule hydrolysis, the hydrolysis efficiency of AmyM-TR2 on 10% raw starch was evaluated. At the enzyme dosage of 0.2 U/mg starch, AmyM-TR2 achieved exceeding 70% DH for 10% NCS after 8 h, while the DH of AmyM was only 14% (Fig. 1A). For both 10% NWS and NCS, AmyM-TR2 achieved a degree of hydrolysis comparable to AmyM (Fig. 1B), even with an enzyme mass (mg) over 30-fold lower (0.025 vs 0.4 U/mg starch, respectively). These results revealed that AmyM-TR2 possessed an unconventional high hydrolysis efficiency for starch granules, indicating the superior potential for application in raw starch processing.

### Binding disparities of enzymes to starch granules

To elucidate the anomalously high hydrolysis efficiency of AmyM-TR2 on starch granules, the binding behaviors of enzymes to starch granules were investigated. AmyM displayed intense fluorescence aggregation at granule centers in fluorescence-based binding assays (Fig. S4), contrasting with the typical localization of other RSDEs at granule edges or pores (9, 18). In comparison, AmyM-TR2 demonstrated dispersive binding to the surface regions of starch granules, in addition to the central aggregation.

**Fig 2:**
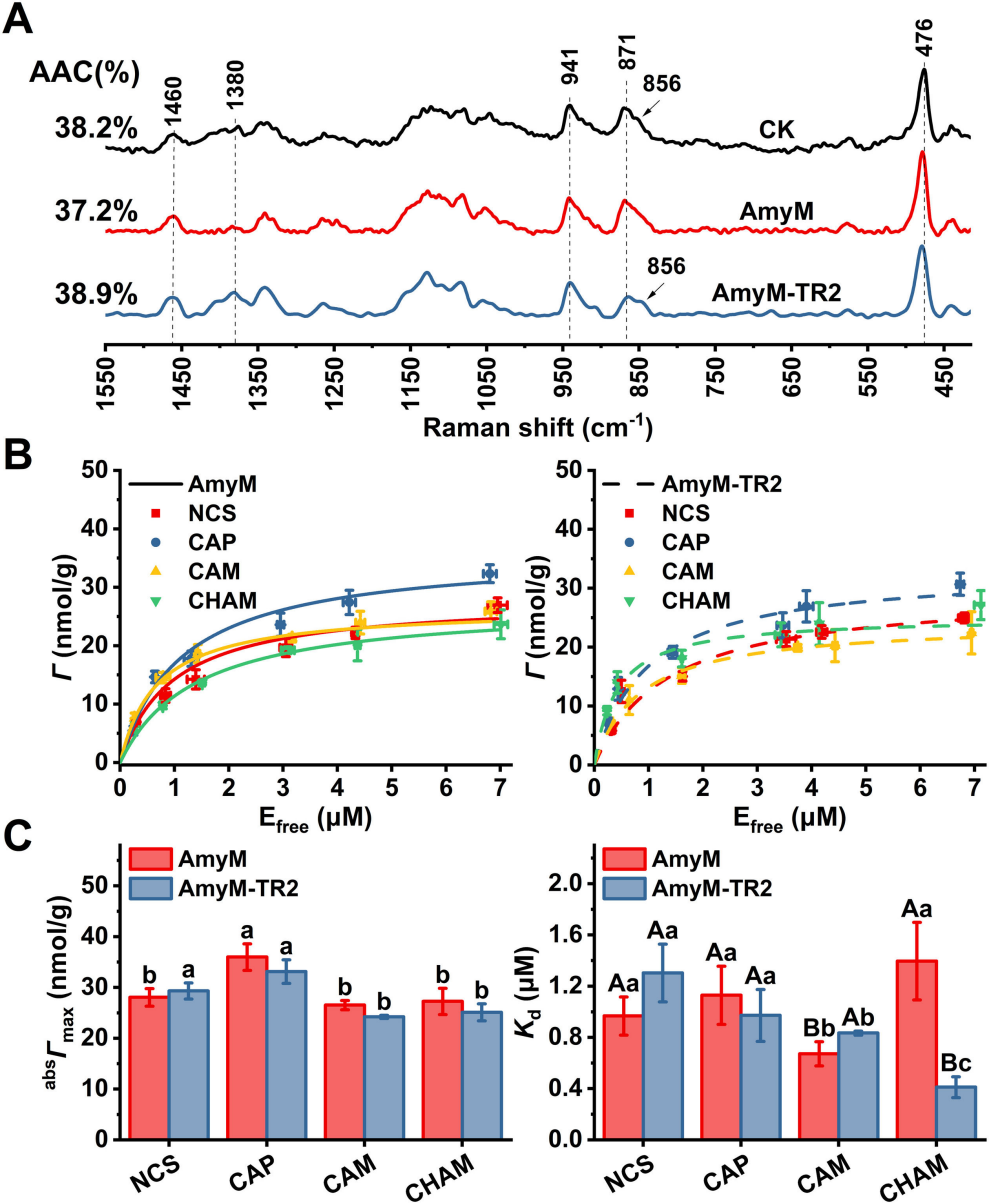
The binding behaviors of AmyM and AmyM-TR2 toward starch granules. (**A**) Apparent amylose content (AAC) and Raman spectra of normal corn starch (NCS) granules treated by AmyM and AmyM-TR2. (**B**) Binding isotherms for AmyM and AmyM-TR2 on NCS, amylopectin (CAP), amylose (CAM), and high-amylose corn starch (CHAM). Lines represent the best fit of the Langmuir equation 2. (**C**) Binding parameters (^ads^*Γ*_max_ and *K*_d_) obtained from binding isotherms. Different uppercase letters indicate significant differences between enzymes, while lowercase letters indicate significant differences between substrates (*P* < 0.05).

The 800–1,600 cm^−1^ region in Raman spectra of starch is referred to as the glucan fingerprint. Bands at 1,420 cm^−1^ were associated with C-H and CH₂ bending modes, while bands at 1,380 cm^−1^ were assigned to C-C-H and C-O-H deformation modes. Key Raman marker bands for different starch molecules had been assigned (23), with bands at 856 and 941 cm^−1^ corresponding to amylose (α-1,4 linkages) and the band at 871 cm^−1^ associated with amylopectin (α-1,6 branching). Raman spectra and apparent amylose content analysis demonstrated that AmyM and AmyM-TR2 exhibited distinct hydrolysis preferences toward starch molecules during the initial hydrolysis stages (Fig. 2A). The key amylose band at 856 cm^−1^ disappeared in AmyM-treated starch, accompanied by a marked decrease in the band intensity at 1,380 cm^−1^. In contrast, starch treated by AmyM-TR2 retained the band at 856 cm^−1^ but exhibited a significant reduction in the hallmark amylopectin band at 871 cm^−1^. Consistent with Raman observations, the AAC decreased after AmyM treatment but increased following AmyM-TR2 treatment. These results suggested that the removal of CBM20 in AmyM altered its binding sites on starch granules, accompanied by a shift in hydrolysis preference from amylose to amylopectin.

The approximate values of the apparent saturation coverage of enzymes enabled the estimation of binding site density (^ads^*Γ*_max_) of AmyM and AmyM-TR2 on different granular surfaces (Fig. 2B). No significant differences in binding site density (^ads^*Γ*_max_) were observed between AmyM and AmyM-TR2 across starch substrates, with both enzymes demonstrating maximum ^ads^*Γ*_max_ values on CAP (36.0 and 33.1 nmol/g, respectively) and minimum ^ads^*Γ*_max_ values on CAM (26.5 and 24.2 nmol/g, respectively) (Fig. 2C). However, CBM20 removal in AmyM increased the binding affinity (1/*K*_d_) for CHAM by 3.4-fold, demonstrating improved affinity to binding sites in starch granules. These results suggested that despite possessing distinct high-affinity binding sites, AmyM-TR2 did not achieve a higher binding site density on starch granules.

### Sequence analysis and molecular docking with double-helical α-glucan molecules

**Fig 3:**
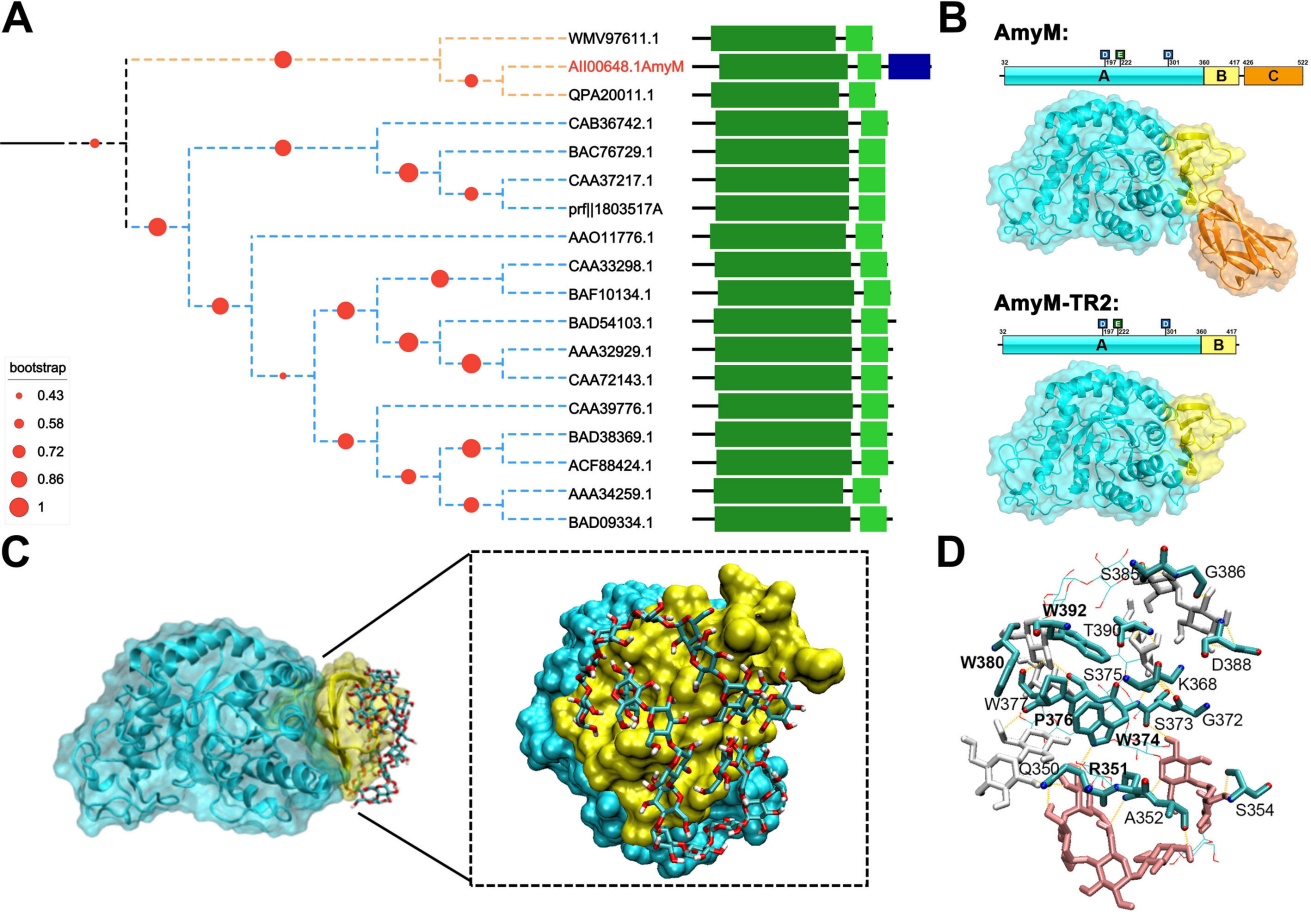
Sequence analysis and molecular docking of Amym-TR2 with double-helical α-glucan molecules. (**A**) Evolutionary tree of the subfamily GH13_6. Orange dashed line: source of bacteria, blue dashed line: source of plant. Dark green square: α-amylase catalytic domain containing domains A and B; lime green square: C-terminal β-sheet domain, domain C; blue square: CBM20 domain. (**B**) Structural modeling and domain architecture of AmyM and AmyM-TR2. Schematics include the three catalytic acids Asp197, Glu222, and Asp301. (**C**) Molecular docking and (**D**) corresponding hydrogen bonding network of AmyM-TR2 with double-helical α-glucan molecules. Side chains involved in hydrogen bonding are shown in teal sticks with nitrogens indicated in blue and oxygens in red. Hydrogen bonds are indicated by yellow dashed lines.

GH13_6 subfamily primarily comprises α-amylases originating from higher plants and green algae. Compared to other GH13_6 subfamily members (Fig. 3A), AmyM featured an additional starch-binding domain (CBM20) alongside the conserved catalytic (β/α)₈-barrel domain and C-terminal β-sheet domain (domain C). Removal of CBM20 in AmyM resulted in more extensive exposure of domain C (Fig. 3B). As a typical RSDE from the GH13_6 subfamily, domain C in AMY1 had been shown to specifically recognize and bind amylopectin via SBS2 in the degradation of starch granules (18).

The crystalline layer of starch granules is composed of double-helical structures formed by amylopectin side chains, contributing to the resistance to enzymatic hydrolysis. Consistent with the binding behaviors to starch granules, AmyM-TR2 treatment had been shown to cause more severe disruption of double helices of starch molecules in fermented dough compared to AmyM treatment (11). We therefore speculated that removal of CBM20 in AmyM unmasked a latent SBD, which promoted the targeted binding and hydrolysis of double helices of amylopectin molecules within starch granules. Molecular docking analysis revealed that double-helical α-glucan molecules could tightly bind to domain C (rather than the catalytic domain) of AmyM-TR2 through hydrogen-bonding networks and hydrophobic interactions. The groove-shaped pocket of domain C perfectly accommodated double-helical α-glucan with appropriate dimensional compatibility (Fig. 3C). Enriched aromatic amino acids (e.g., tryptophan) and polar residues within the pocket formed potential hydrogen bonds with the chain of double-helical α-glucan in the complex structure (Fig. 3D).

The effect of domain C and CBM20 domain on interactions with substrates of varying conformations was further investigated, including linear maltododecaose (G12), double-helical α-glucan, and amylose helix-mimicking β-cyclodextrin (20). The binding energies of AmyM with G12, double-helical α-glucan, and β-cyclodextrin were −6.7, −4.6, and −8.5 kcal/mol, respectively (Table S3). AmyM-TR2 exhibited a lower binding energy (−5.3 kcal/mol) with the double helix molecule, while its binding energies with G12 and β-cyclodextrin increased to −6.5 and −7.0 kcal/mol, respectively. These results indicate that the CBM20 domain in AmyM played a crucial role in binding to linear and single-helical starch chains (amylose), while the exposed domain C facilitated interaction with double-helical molecules (amylopectin).

### Interfacial enzyme kinetics mediated by RSDE-specific productive binding sites

To validate the binding preference of AmyM-TR2 for amylopectin double helices, catalytic kinetic analysis was performed using gelatinized and granular corn starches with varying amylose contents as substrates, respectively. In the kinetic analysis of gelatinized starch, CBM20 removal in AmyM led to a 22.2%–67.6% reduction in *k*_cat_ values toward all substrates yet was accompanied by a general increase in substrate affinity (Table S4). The greatest enhancement in affinity was observed for CAP (a 2.57-fold increase), followed by CHAM (2.22-fold), PSS (1.53-fold), NCS (1.46-fold), and CAM (1.18-fold). AmyM-TR2 exhibited a 2.02-fold increase in catalytic efficiency (*k*_cat_/*K*_m_) toward CAP compared to AmyM while showing significantly reduced efficiency toward other substrates, most notably for CAM (0.59-fold). These results indicated that the truncation of CBM20 significantly enhanced the catalytic efficiency toward amylopectin through improved affinity but reduced catalytic efficiency toward amylose.

**Fig 4:**
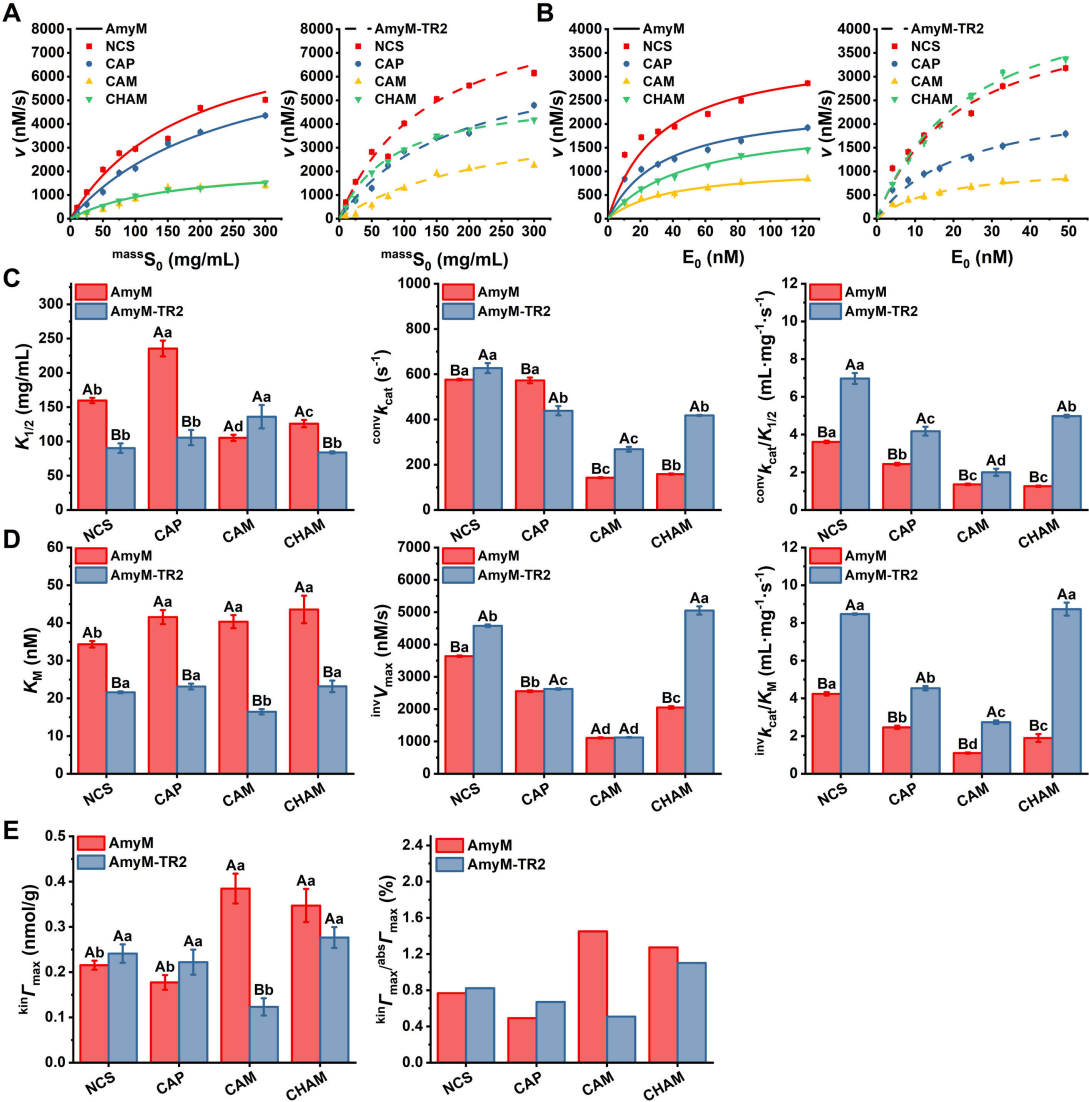
Interfacial catalysis of various starch granules by AmyM and AmyM-TR2 at 50°C and pH 7. (**A**) Conventional and (**B**) inverse kinetics for AmyM and AmyM-TR2 on normal corn starch (NCS), amylopectin (CAP), amylose (CAM), and high-amylose corn starch (CHAM). Lines in parts A and B are fit using equations 4 and 5, respectively. (**C**) Kinetic parameters (*K*_1/2_, ^conv^*k*_cat_, and ^conv^*k*_cat_/*K*_1/2_) obtained from CMM kinetics. (**D**) Kinetic parameters (*K*_M_, ^inv^*V*_max_, and ^inv^*k*_cat_/*K*_M_) obtained from IMM kinetics. (**E**) ^kin^*Γ*_max_ (attack site density) and ^kin^*Γ*_max-ave_/^abs^*Γ*_max-ave_ for AmyM and AmyM-TR2 on starch granule substrates obtained from equation 7. Different uppercase letters indicate significant differences between enzyme mutants, while lowercase letters indicate significant differences between substrates (*P* < 0.05).

Interfacial kinetics of various granular starch substrates were investigated through analysis of CMM (Fig. 4A) and IMM (Fig. 4B) kinetic curves. All *K*_1/2_ and *K*_M_ values fell within experimentally tested concentration ranges of granular starch substrates and enzymes. AmyM and AmyM-TR2 exhibited distinctly different interfacial catalytic properties toward starch granules compared to reported glucoamylase GA and psychrophilic α-amylase AHA (8, 9).

In CMM kinetic analysis, AmyM showed the lowest *K*_1/2_ value for CAM (105.2 mg/mL), followed successively by CHAM (125.9 mg/mL), NCS (159.6 mg/mL), and CAP (235.5 mg/mL) (Fig. 4C). In contrast, AmyM-TR2 displayed 1.8-, 2.2-, and 1.5-fold reductions in *K*_1/2_ values for NCS, CAP, and CHAM, respectively, but showed a higher *K*_1/2_ value for CAM (136.0 mg/mL). Meanwhile, the ^conv^*k*_cat_/*K*_1/2_ values of AmyM-TR2 were improved by 1.93-, 1.72-, 1.47-, and 3.95-fold for NCS, CAP, CAM, and CHAM, respectively. In the IMM kinetic analysis, AmyM-TR2 exhibited 1.5- to 2.5-fold lower *K*_M_ values across all substrates compared to AmyM (Fig. 4D). Consistent with CMM kinetics, AmyM-TR2 displayed a 1.84- to 4.59-fold increase in ^inv^*k*_cat_/*K*_M_ values across the substrates. AmyM demonstrated the highest attack site density (^kin^*Γ*_max_) on CAM (0.38 nmol/g), followed by CHAM (0.35 nmol/g), NCS (0.22 nmol/g), and CAP (0.18 nmol/g), correlating with substrate amylose content (Fig. 4E). In contrast, AmyM-TR2 showed the minimal ^kin^*Γ*_max_ value on CAM (0.12 nmol/g) but comparable values (0.22–0.28 nmol/g) for NCS, CAP, and CHAM. It was noteworthy that the differences in affinity and attack site density between AmyM and AmyM-TR2 for various granular substrates exhibited a discernible correlation with substrate amylopectin content. These results supported that CBM20 removal in AmyM led to a shift in substrate targeting from amylose to amylopectin in starch granules.

Attack sites of enzymes often correspond closely to productive binding sites (PBSs) of substrates. The concentration of PBSs available for enzymatic binding and hydrolysis reflected the productive binding capacity of the substrate in the interfacial enzyme reaction of cellulose (24). However, this was not applicable to the interfacial enzyme reaction of starch granules. AmyM exhibited the lowest catalytic efficiency toward CAM, which possessed the highest attack site density and affinity. Similar results were observed in the interfacial catalytic reaction of GA on WMS (9). AmyM and AmyM-TR2 possessed lower affinity and attack site density toward starch granules but maintained high catalytic efficiency. Both enzymes exhibited remarkably low ^kin^*Γ*_max-ave_/^abs^*Γ*_max-ave_ values across substrates (0.5%–1.3%). We therefore hypothesized that various starch granules possessed multiple PBSs with distinct hydrolysis properties, and their interfacial catalytic kinetics predominantly reflect the catalytic characteristics of enzymes interacting with the targeted PBSs. Combined with the starch granule binding results, the altered substrate targeting conferred novel high-affinity PBSs in the binding and hydrolysis of AmyM-TR2 for starch granules, thereby enhancing catalytic efficiency.

High-amylose starch is considered highly resistant to enzymatic hydrolysis (25). However, AmyM-TR2 exhibited unexpectedly high catalytic efficiency and distinct interfacial catalytic kinetics toward CHAM. In CMM kinetics, the kinetic characteristics of AmyM-TR2 for CHAM were comparable to those of CAP, whereas AmyM showed overlapping kinetic curves toward CHAM and CAM. These results suggested that preferential binding of AmyM to amylose constrained its catalytic efficiency toward CHAM, whereas AmyM-TR2 efficiently targeted and hydrolyzed the amylopectin fraction in CHAM.

Nill and Jeoh reported that the productive binding capacity governed the initial hydrolysis rate in interfacial enzyme reactions, whereas the depletion of PBSs explained overall hydrolysis kinetics (6). The hydrolysis curves of 10% NCS revealed that AmyM reached a plateau phase within 1 h, while AmyM-TR2 maintained efficient hydrolysis for up to 8 h before attaining equilibrium (Fig. 1A). This result indicated that the targeted PBSs of AmyM-TR2 exhibited significantly higher sustained hydrolyzability compared to those of AmyM. AmyM-TR2 exhibited high catalytic efficiency toward both NCS and CHAM with similar IMM kinetic curves, suggesting that its targeted PBSs in multi-component starch possessed sustained hydrolyzability.

### Efficient hydrolysis of starch granules by direct interactions with amylopectin double helices via domain C

**Fig 5:**
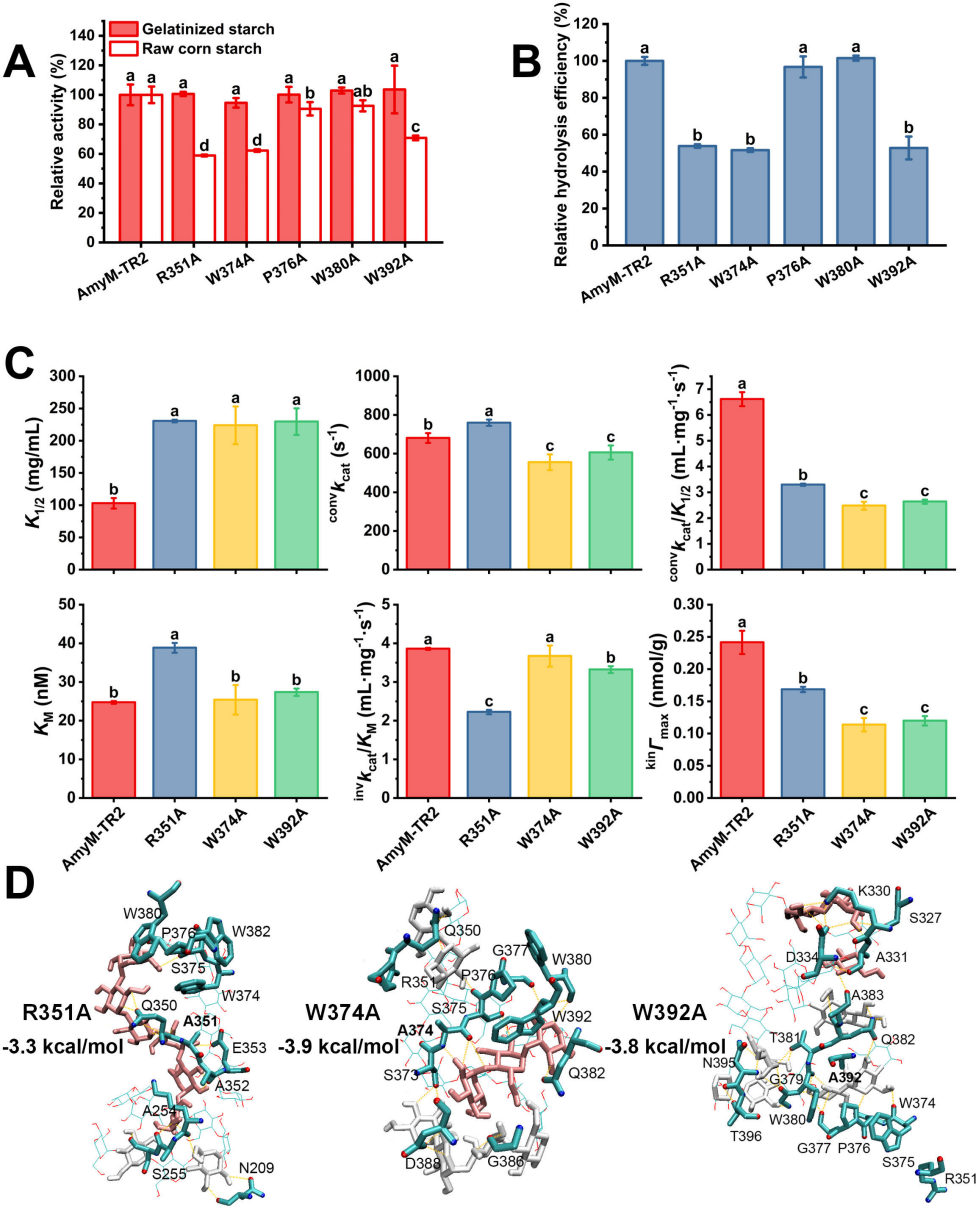
Role of key amino acid residues involved in double-helix recognition in starch granule hydrolysis by AmyM-TR2. (**A**) Relative activity of AmyM-TR2 and its mutants toward gelatinized and granular starch. The assay of enzymatic activity against gelatinized and granular starch substrates was performed under optimum conditions with 5 mg/mL soluble starch and 20 mg/mL normal corn starch, respectively. (**B**) Relative hydrolysis efficiency of AmyM-TR2 and its mutants toward 10% NCS with an enzyme dosage of 0.2 U/mg starch. (**C**) Interfacial kinetic parameters (*K*_1/2_, ^conv^*k*_cat_, ^conv^*k*_cat_/*K*_1/2_, *K*_M_, ^inv^*k*_cat_/*K*_M_, and ^kin^*Γ*_max_) of AmyM-TR2 mutants for amylopectin. (**D**) Corresponding hydrogen bonding network and binding energy of R351A, W374A, and W392A mutants with double-helical α-glucan molecules. Different lowercase letters indicate significant differences between enzymes (*P* < 0.05).

The binding ability of most CBMs and SBSs is primarily mediated by aromatic residues, which interact with sugar rings through CH–π interactions; in addition, basic residues (e.g., lysine, arginine, and histidine) can also contribute to polysaccharide binding via electrostatic interactions and hydrogen bonding (26). Therefore, we reasoned that mutation of R351, W374, P376, W380, or W392 to alanine would significantly reduce or abolish binding. All mutants exhibited comparable activity to Amym-TR2 on gelatinized starch (Fig. 5A), suggesting that these sites did not influence the hydrolysis of gelatinized starch. Using granular starch as the substrate, we found that only the R351A, W374A, and W392A mutants exhibited a reduction in enzymatic activity by 29.2%–41.1% (Fig. 5A). At the same enzyme dosage (0.2 U/mg starch), these three mutants exhibited a significant reduction in hydrolysis efficiency toward 10% NCS, with a decrease ranging from 46.1% to 48.3% (Fig. 5B). These results indicated that residues R351, W374, and W392 in domain C played crucial roles in granular starch hydrolysis.

To further confirm the functional roles of these residues, interfacial kinetics for R351A, W374A, and W392A mutants of AmyM-TR2 were analyzed using granular amylopectin as the substrate (Fig. S5). R351A, W374A, and W392A mutants displayed reduced affinity (1/*K*_1/2_) by ~2.2-fold and attack site density (^kin^*Γ*_max_) by 1.4- to 2.1-fold (Fig. 5C). The corresponding catalytic efficiency (^conv^*k*_cat_/*K*_1/2_) of R351A, W374A, and W392A decreased by 2.0-, 2.7-, and 2.5-fold, respectively. IMM kinetic analysis revealed the highest *K*_M_ value for R351A (38.8 nM) toward granular amylopectin, whereas W374A (25.3 nM) and W392A (27.3 nM) shared comparable *K*_M_ values with AmyM-TR2 (24.7 nM). Correspondingly, the R351A mutant demonstrated the lowest ^inv^*k*_cat_/*K*_M_ value (2.22 mL/mg/s), followed by W392A (3.32 mL/mg/s) and W374 (3.67 mL/mg/s). These results demonstrated that mutations at residues R351, W374, and W392 significantly impaired the affinity toward amylopectin, supporting that the efficient hydrolysis performance of AmyM-TR2 was achieved through targeted hydrolysis of amylopectin double helices.

Combined with molecular docking results, the guanidinium group of R351 contributed to the formation of three hydrogen bonds, while the indole rings of W374 and W392 synergistically stabilized the complex through hydrophobic packing and auxiliary hydrogen bonding (Fig. 3D). Alanine substitutions at residues R351A, W374A, and W392A disrupted critical interactions (Table S5), thereby weakening the binding capacity (1.4–2.0 kcal/mol) to double-helical α-glucan chains (Fig. 5D). The R351A mutant exhibited a 67% reduction in hydrogen bonding capacity with the highest binding energy (−3.1 kcal/mol). The order of binding energy penalties was consistent with the corresponding ^inv^*k*_cat_/*K*_M_ values (R351A > W392A > W374A). These results indicated that the residues R351, W374, and W392 in domain C contributed to the catalytic specificity of AmyM-TR2 toward starch granules by direct interactions with amylopectin double helices.

### Universal role of domain C in RSDEs

The α-amylase family exhibits a highly conserved tertiary structure comprising three domains (A, B, and C) (27). Domain C adopts an antiparallel β-sheet configuration, typically positioned at the C-terminus, though its function remains unknown. The truncation of domain C in the RSDE Gt-amyII abolished starch granule adsorption and hydrolysis abilities (28), indicating the important role of domain C in CBM-lacking RSDEs. Considering the function of domain C in AmyM-TR2, we suggested that domain C in RSDEs served as potential SBDs.

**Fig 6:**
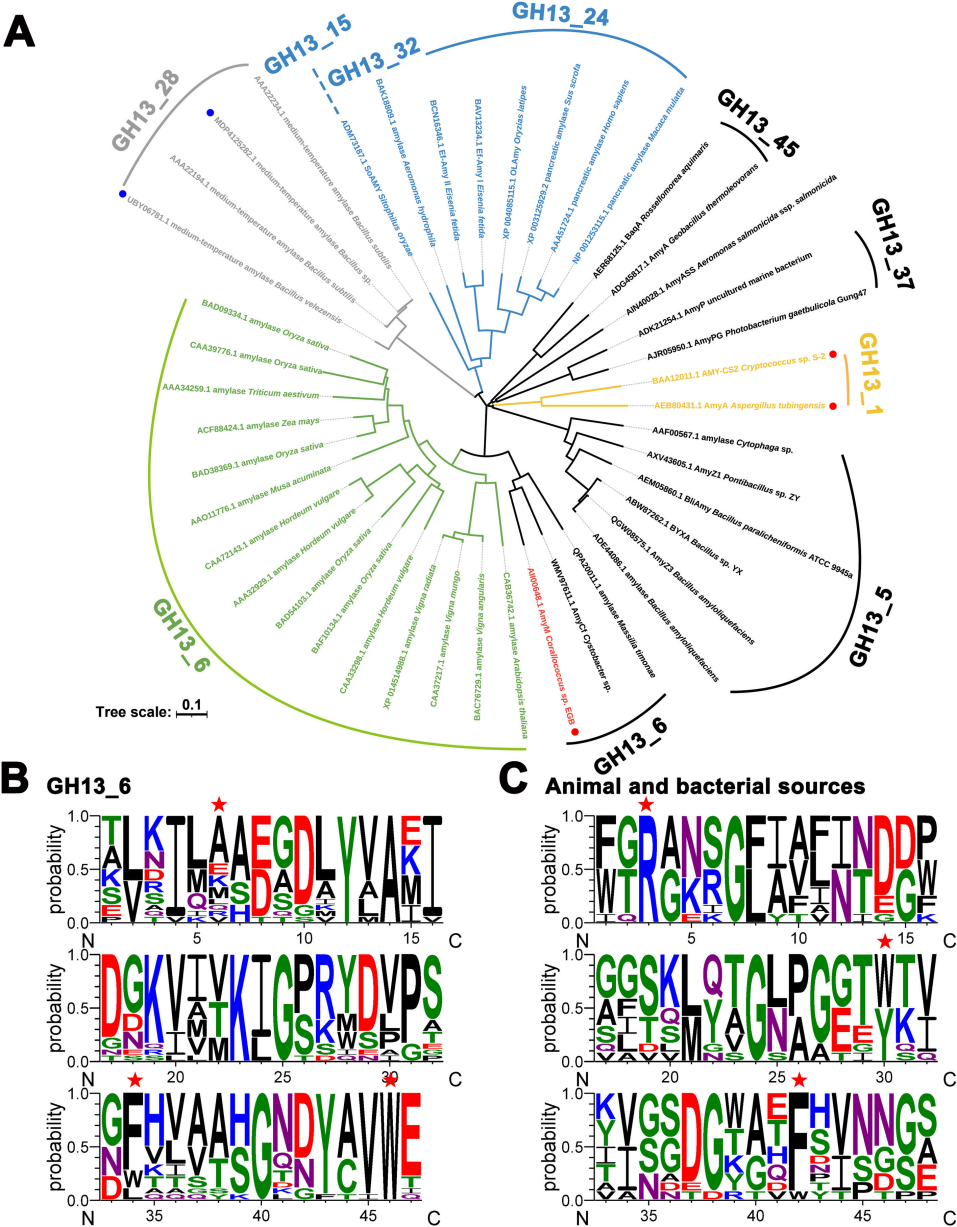
Evolution and amino acid conservation analysis of domain C in RSDEs. (**A**) Evolutionary tree of domain C in RSDEs. The 43 members included GH13_6 subfamily amylases, reported or potential RSDEs, and medium-temperature amylase (as the control). Red circles indicate the presence of the additional CBM20 domain, while blue circles denote the inclusion of CBM26. (**B**) Amino acid conservation analysis of domain C in GH13_6 subfamily members. (**C**) Amino acid conservation analysis of domain C in AmyM and other bacterial- and animal-derived RSDEs. Red stars indicate the equivalent amino acid residues R351, W374, and W392.

The tree based on short conserved sequences from domains A and B respects α-amylase specificity, whereas the phylogenetic tree of domain C exhibits separate evolution, with substantial changes in the arrangement and clustering of individual enzymes and enzyme specificities (29). By characterizing the members of subfamilies of the GH13 family, we have shown that RSDEs are widely distributed in the subfamilies GH13_1, 5, 6, 15, 24, 28, 32, 37, and 45 (Fig. 6A). Comparing the divergent evolutionary trajectories of domain C from microbial-derived RSDEs, domain C of plant-derived RSDEs (from GH13_6), and animal-derived RSDEs (from GH13_15, 32, and 24) formed distinct compact clusters. Within the GH13_6 subfamily, an independent cluster was distinct from other plant members, including AmyM and AmyCf from myxobacteria, and amylase from *Massilia timonae* (13, 30). This result indicated the special evolutionary pathway of AmyM-derived domain C among RSDEs.

Furthermore, the key residues R351, W374, and W392 in domain C displayed lineage-specific conservation patterns among non-fungal RSDEs (Fig. S6) while remaining non-conserved in medium-temperature amylases (control group). W374 was strictly conserved in bacterial RSDEs but substituted by tyrosine (Y) and phenylalanine (F) in plant- and animal-derived RSDEs, respectively (Fig. 6B and C). W392 was conserved in GH13_6 subfamily enzymes and partially conserved as phenylalanine in other RSDE sources. In contrast, R351 was exclusively conserved in animal- and bacterial-derived RSDEs. These results indicated that residues R351, W374, and W392 might form a relatively conserved SBS in domain C of RSDEs.

Previous evolutionary evidence suggested that these residues involved in raw starch binding might be conserved as a relic from a primordial CBM ancestor in CBM-lacking RSDEs (3). In contrast to non-fungal RSDEs that typically lack CBMs, fungal-derived RSDEs evolved the CBM20 domain while lacking the three conserved residues (R351, W374, and W392) within domain C (Fig. 6A; Fig. S6). Hence, the emergence of CBM20 in RSDEs or the loss of raw starch activity could result from the absence of ancestral raw starch-binding residues.

## DISCUSSION

In this study, the CBM20-truncated mutant AmyM-TR2 demonstrated unexpectedly high efficiency in starch granule hydrolysis, challenging conventional understanding of RSDEs' catalytic properties. The obtained results revealed a novel mechanism for efficient RSDE–starch granule hydrolysis, wherein AmyM-TR2 efficiently hydrolyzed starch granules by targeting and hydrolyzing amylopectin double helices via the exposed domain C. In contrast, the presence of the CBM20 domain in AmyM promoted preferential binding and hydrolysis of amylose on the surface of starch granules. By integrating enzyme substrate-targeting specificity with interfacial catalytic analysis, this study advances the conceptual understanding of how enzyme–substrate matching governs interfacial catalysis on heterogeneous starch granules.

### Widespread existence of starch-binding modules with the capacity of recognizing double-helical molecules

Polysaccharides exert a variety of chain conformations in solution, such as random coil, single helix, double helix, and triple helix (31). Polysaccharide-binding studies have traditionally focused on interactions with linear or branched glucan chains, implicitly assuming that chain conformation plays a secondary role in substrate recognition. However, starch granules are dominated by higher-order conformations, particularly amylopectin double helices that define crystalline lamellae and hydrolysis resistance (32). Only a limited number of studies have examined domains interacting with double-helical molecules (20).

Molecular docking between α-amylase and double-helical molecules was performed by Zhong et al., revealing that double-helical molecules could not dock to the active site of amylase due to steric hindrance (33). In this study, our results demonstrated that AmyM-TR2 could achieve effective starch granule binding through SBSs in domain C, enabling direct recognition of double-helical α-glucans. Similar interactions between SBSs in the non-CBM domain and double-helical molecules had been observed in enzymes involved in starch synthesis and metabolism in plants (34). SBS3 in the C-terminal domain of *Arabidopsis* glucan phosphatase LSF2 has been proven to play a more critical role in amylopectin binding, with the observed binding to helical glucan chains (35). Together, these observations suggest that starch-binding modules capable of recognizing amylopectin double helices might be more widespread than previously appreciated, functioning across starch synthesis, remodeling, and degradation pathways.

### Efficient degradation of insoluble substrates mediated through targeted hydrolysis of molecules with specific conformation

The adsorption of enzymes onto starch granules during hydrolysis was a highly selective process, with most binding sites allowing for enzymatic catalysis (19). Differences in binding site types for adsorption and hydrolysis within insoluble polysaccharides significantly influenced degradation efficiency. The CBM17 and CBM28 domain in cellulase Cel5A recognized different sites on non-crystalline cellulose, exerting distinct effects on enzymatic degradation efficiency (36).

Conventional truncation or proteolytic removal of CBMs generally preserves soluble substrate activity while drastically impairing insoluble substrate degradation (37). As a type B CBM, CBM20 domain possesses groove- or crack-like binding sites for individual glycan chain interactions (38). Combined with the data reported here, the presence of CBM20 in AmyM promoted the preferential binding to amylose on the granule surface, while AmyM-TR2 preferentially cleaved amylopectin double helices. These contrasting binding preferences indicate that differences in the types of productive binding sites targeted within starch granules critically influence degradation efficiency. Contrary to reported findings, the CBM20-truncated mutant AmyM-TR2 exhibited disproportionately high degradation efficiency toward starch granules, highlighting that efficient degradation of insoluble substrates can be achieved through selective targeting of specific conformational motifs rather than indiscriminate enhancement of surface adsorption.

This mechanism provides a conceptual parallel to recent observations in the degradation of other crystalline polymers, including cellulose and synthetic plastics, where disruption of ordered domains rather than bulk adsorption governs overall depolymerization efficiency (39, 40). Accordingly, engineering strategies that prioritize conformation-specific targeting could offer a more effective route to improving enzymatic degradation of insoluble substrates than indiscriminate enhancement of surface binding.

### Novel insights into interfacial catalytic mechanisms from diverse RSDE–PBS interactions

Current research on interfacial catalysis of insoluble substrates primarily used structurally homogeneous cellulose as a model substrate to derive parameters such as attack site density, without adequately accounting for the potential complexity of binding sites in other substrates (5–7). However, starch granules represent a fundamentally different class of insoluble substrates characterized by compositional and conformational heterogeneity at the surface. Accordingly, the applicability and interpretation of interfacial kinetic parameters for starch granules remain insufficiently explored. Here, this study extends interfacial kinetic analysis by explicitly linking enzyme substrate-targeting specificity with the heterogeneous nature of productive binding sites (PBSs) in starch granules.

**Fig 7:**
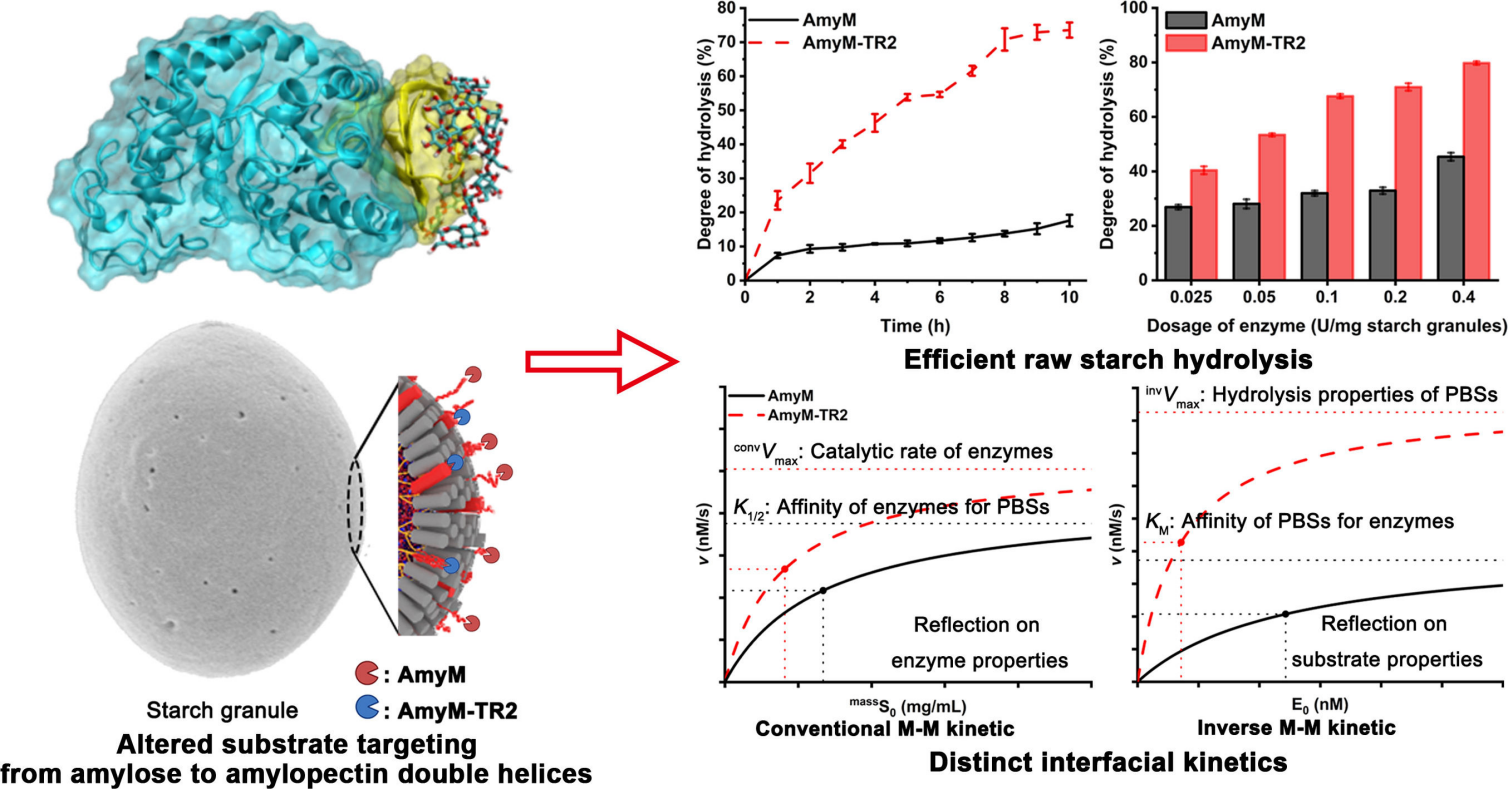
Targeted hydrolysis of amylopectin double helices for efficient raw starch hydrolysis. Using high-amylose corn starch (CHAM) as a substrate, the surface structural model of CHAM was modified from reference 41 with permission of the publisher. Red lines represent amylose chains, and red cylinders denote amylopectin double helices. AmyM and AmyM-TR2 specifically target amylose and amylopectin double helices in the granule surface, respectively. The binding of enzymes to different conformational molecules represents their interaction with PBSs with different hydrolysis characteristics, exhibiting distinct degradation processes and interfacial catalytic kinetics. While conventional M-M kinetics reflect enzymatic catalytic properties, inverse M-M kinetics characterize the properties of PBSs within substrates.

Based on the results obtained in this study, we proposed that the IMM analysis provided a characterization of PBSs targeted by enzymes in interfacial enzyme reactions (Fig. 7). Interfacial kinetic parameters *K*_1/2_ and *K*_M_ values reflected the affinity between enzyme and preferentially bound PBSs, whereas ^kin^*Γ*_max_ values corresponded to the abundance of such PBSs (Fig. 4). Importantly, the hydrolysis processivity of targeted PBSs represented an additional determinant of sustained interfacial catalysis that was not explicitly captured in conventional kinetic interpretations.

Extensive research has characterized the surface of various starch granules, exhibiting distinct differences between single-component and multi-component starch. The surface of single-component starch may be structurally simpler. The surface of pure amylose lacked exposure of amylopectin double helices, while the surface of pure amylopectin primarily consisted of double helices formed by the long chains of amylopectin (41). For multi-component starch, structural complexity facilitated the exposure of amylopectin double helices with increased outer A-chain content on the surface (42), suggesting a greater diversity of binding site types. Under enzyme-supersaturated hydrolysis conditions, the *V*_max_ was determined by the total number of PBSs and their hydrolysis processivity. The difference in ^inv^*V*_max_ values toward substrates was influenced by the hydrolysis processivity of their targeted PBSs, implying the complexity of multi-component starch conferred superior hydrolysis processivity of PBSs compared to single-component starch.

We hypothesized that during multi-component starch hydrolysis, the reaction of AmyM-TR2 toward specific targeted PBSs exhibited significantly greater catalytic persistence compared to AmyM (Fig. 7). This catalytic difference (^inv^*V*_max_) was evident in their degradation efficiency of 10% NCS and interfacial catalytic kinetics toward CHAM (Fig. 1 and 4). Overall, interfacial catalytic kinetics emerge as a diagnostic framework for deciphering enzyme–PBS matching in heterogeneous insoluble substrates and guiding the development of high-efficiency interfacial enzymes.

### Conclusion

This study reveals that the exposed domain C endowed the targeting-hydrolysis ability to amylopectin double helices, thereby significantly enhancing the hydrolysis efficiency of starch granules. Three key residues (R351, W374, and W392) in domain C are involved in the binding of double-helical molecules, displaying lineage-specific conservation patterns across RSDEs. The comparative analysis of interfacial kinetics of AmyM-TR2 and wild-type AmyM toward different starches confirms distinct substrate-targeting specificities and variations in their targeted PBSs, providing new insights for applying interfacial catalytic kinetics and engineering efficient interfacial enzymes. We propose that the persistent catalysis capacity of interfacial enzymes to target specific conformational molecules involved in substrate crystallinity is key to achieving efficient degradation of insoluble substrates. The efficient catalytic mechanism of AmyM-TR2 provides a valuable framework for informing interfacial catalytic kinetics and interfacial enzyme engineering.

## SUPPLEMENTAL MATERIAL

Supplemental material aem.01908-25-s0001.doc Fig. S1 to S6; Tables S1 to S5.

ASM does not own the copyrights to Supplemental Material that may be linked to, or accessed through, an article. The authors have granted ASM a non-exclusive, world-wide license to publish the Supplemental Material files. Please contact the corresponding author directly for reuse.
