# Supplementary material for: Mechanistic insights into substrate-targeting modification effects on interfacial kinetics of raw starch-degrading amylases
Source: Appl Environ Microbiol. 2026 Apr 6;92(5):e01908-25. doi: 10.1128/aem.01908-25 (PMC13188927; doi:10.1128/aem.01908-25)
Supplement: Supplemental material — Fig. S1 to S6; Tables S1 to S5. [file aem.01908-25-s0001.doc]

Supporting Information

**Mechanistic insights into substrate-targeting modification effects on interfacial kinetics of raw starch-degrading amylases**

Lingli Zhong ^a^, Zongchao Huo ^b^, Min Jiang ^a^, Wenming Chang ^a^, Kai Yang ^a^, Xue Chen ^a^, Xianfeng Ye ^a^, Yanling Ji ^a^, Yan Huang ^a^, Lei Zhang ^a^, Zhoukun Li ^a*^, Yanwei Li ^b*^, Zhongli Cui ^a*^


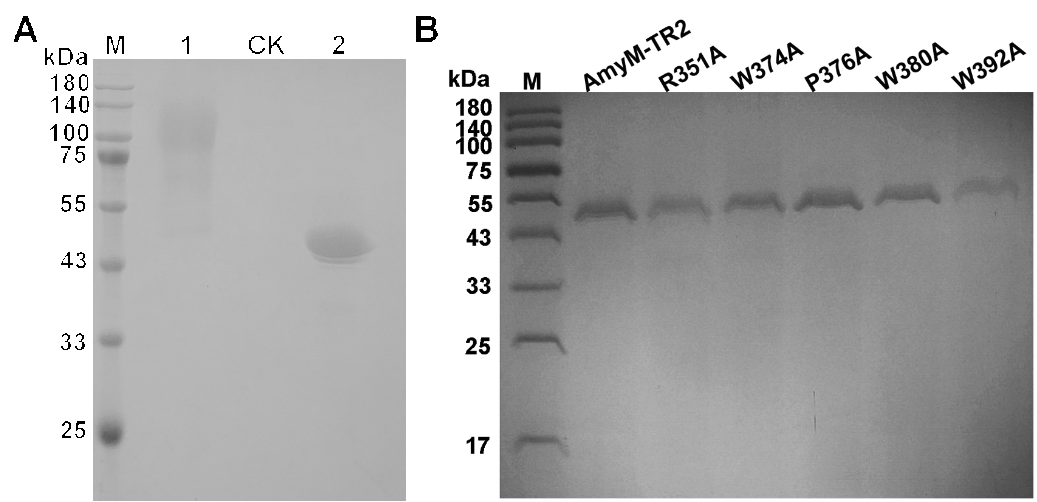


**Fig. S1. SDS-PAGE analysis of AmyM-TR2 and its mutants.** (A) SDS-PAGE analysis of purified AmyM and AmyM-TR2. Lane M, standard protein molecular mass marker; line CK, the fermentation supernatant of *P. pastoris* GS115 harboring the vector pEFαA; lane 1, AmyM; lane 2, AmyM-TR2. (B) SDSPAGE analysis of purified AmyM-TR2 mutants.


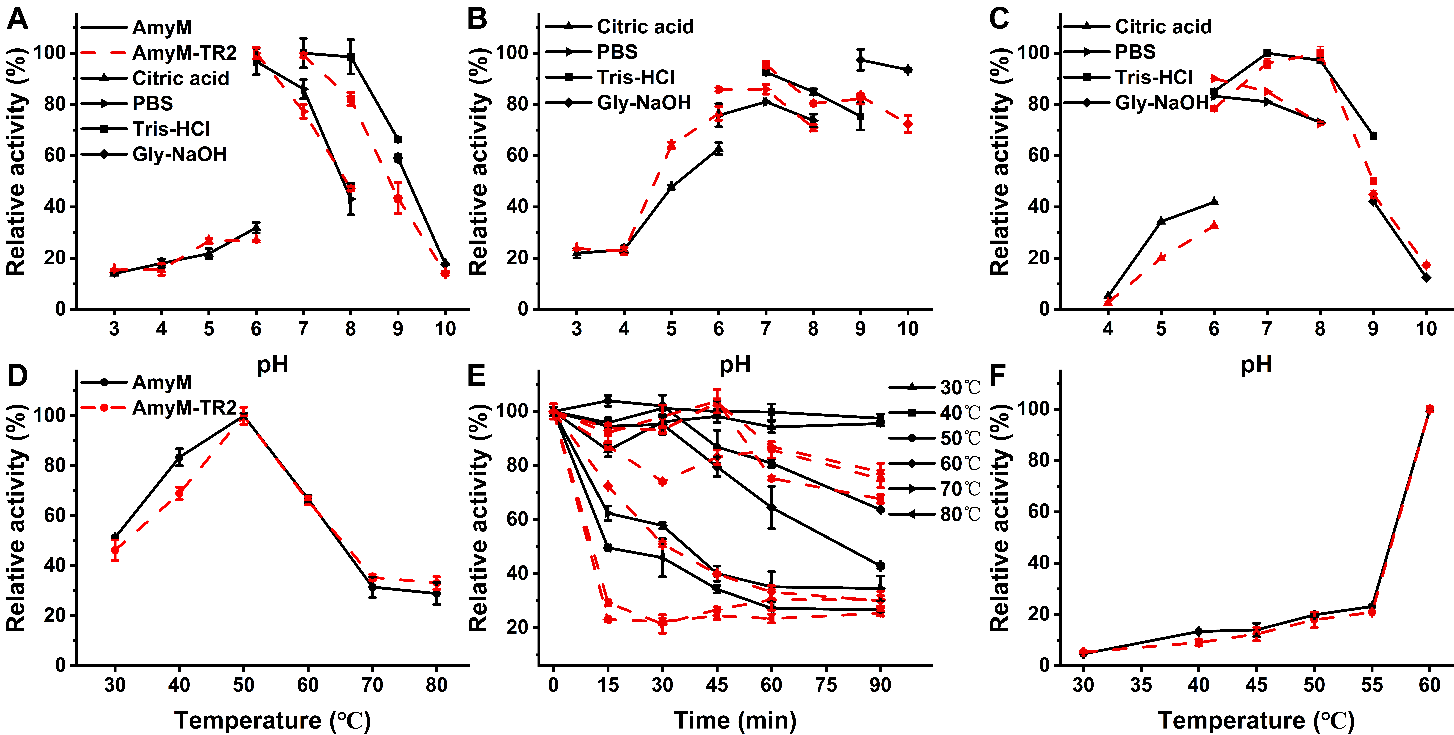


**Fig. S2. Biochemical properties of AmyM-TR2.** (A, B) Effect of pH on the enzyme activity and stability of AmyM-TR2 toward gelatinized soluble starch. (D, E) Effect of temperature on the enzyme activity and stability of AmyM-TR2 toward gelatinized soluble starch from 30 ℃ to 80 ℃. (C, F) Effect of pH and temperature on the enzyme activity of AmyM-TR2 toward wheat starch granules.


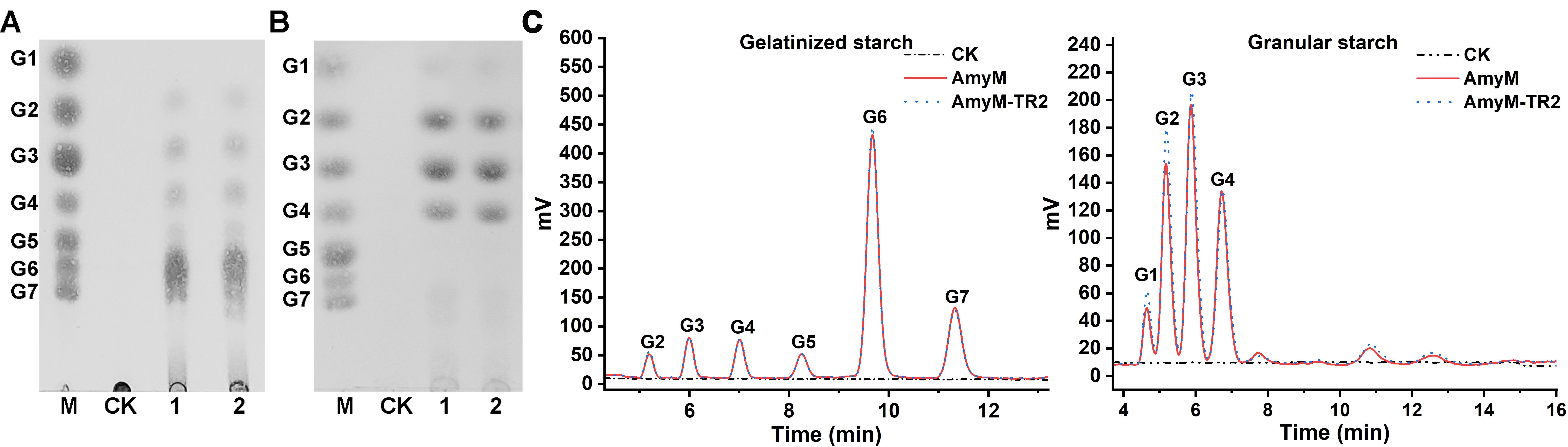


**Fig. S3. TLC and HPLC analysis of the hydrolysis products of AmyM and AmyM-TR2.** (A) TLC analysis of the hydrolysis products of gelatinized soluble starch hydrolyzed by AmyM and AmyM-TR2. (B) TLC analysis of the hydrolysis products of wheat starch granules hydrolyzed by AmyM and AmyM-TR2. Lane M, standard malto-oligosaccharide mixture from glucose (G1) to maltoheptaose (G7); lane 1, hydrolysis products of AmyM; lane 2, hydrolysis products of AmyM-TR2. (C) HPLC analysis of the hydrolysis products from gelatinized soluble starch and wheat starch granules.


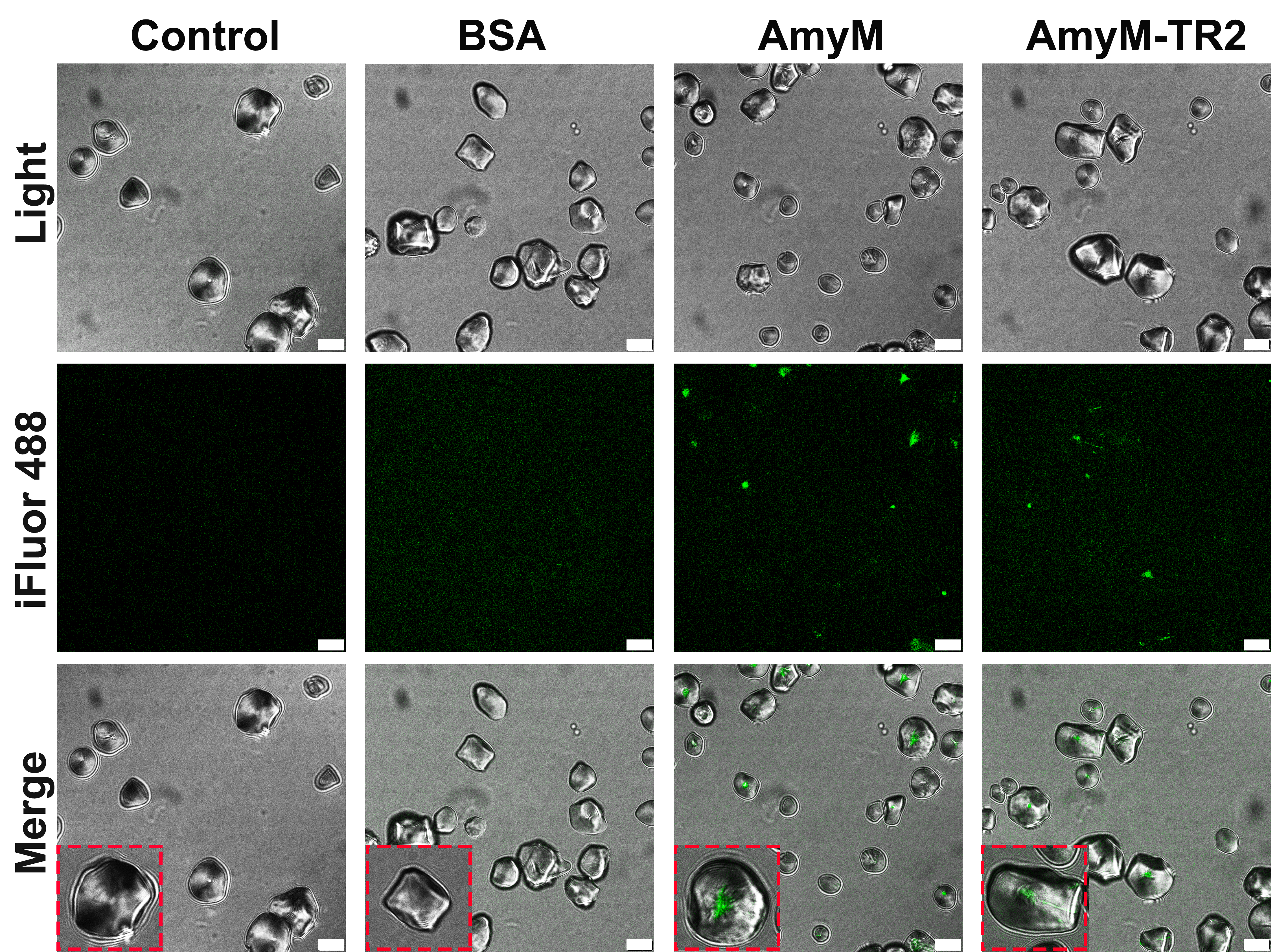


**Fig. S4. Interaction of AmyM and AmyM-TR2 toward normal corn starch granules visualized by CLSM (Scale bar: 10 μm).**

**Fig. S5. Interfacial catalysis of amylopectin granule degradation by AmyM-TR2 and its mutants (R351A, W374A and W392A) at 50 ℃ and pH 7.** (A) CMM and (B) IMM kinetics. Lines in part A and B are fits using equations 3 and 4, respectively.


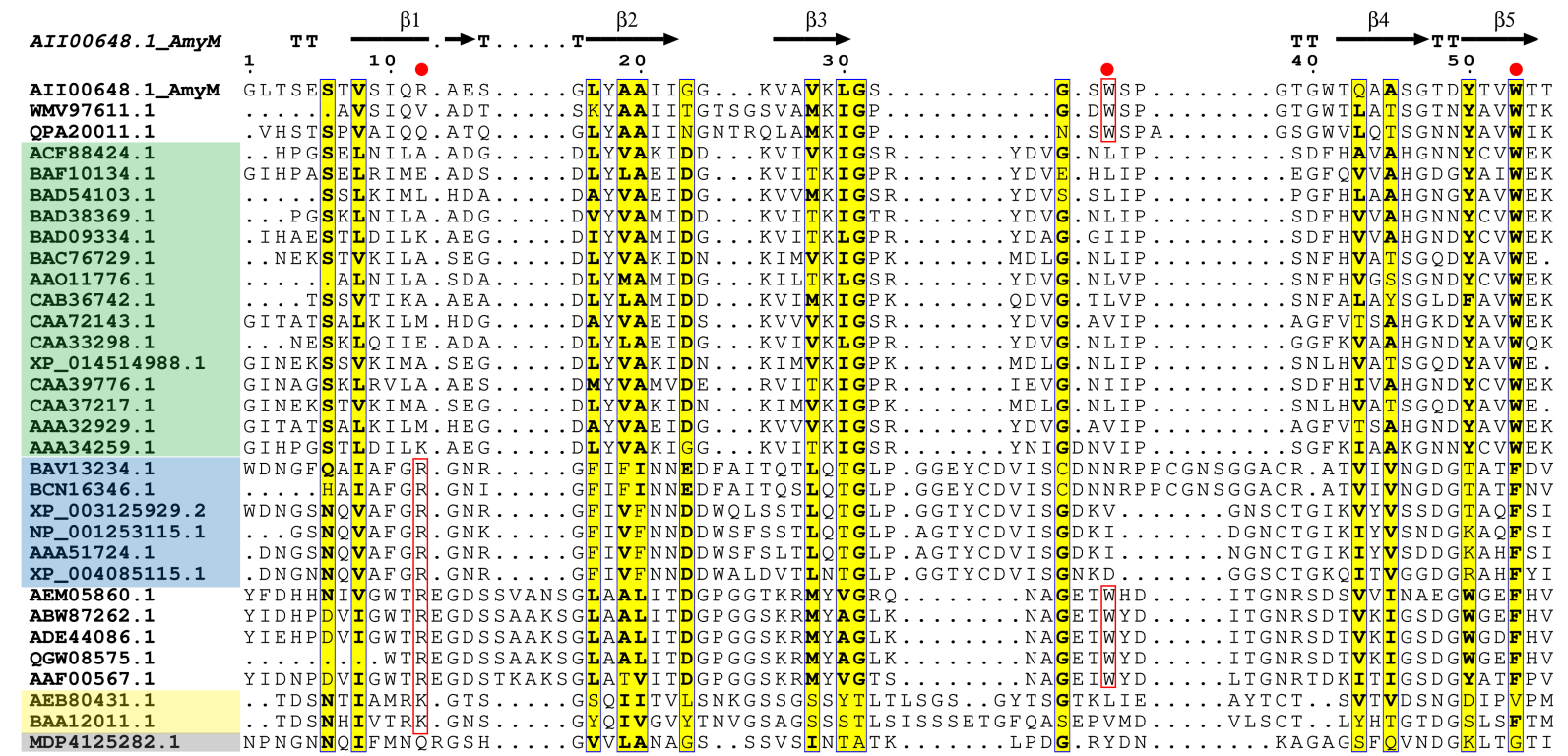


**Fig. S6. Sequence alignment of domain C in RSDEs.** The 32 amylase sequences are selected from the members of the domain C evolutionary tree. The source of enzyme: plant, green; animal, blue; bacteria, blank; fungus, yellow; medium-temperature amylase (as the control), grey.

**Table S1 Primers for mutations in this study.**

| Gene name | Sequence of the primer (5’-3’) |
| --- | --- |
| R351A for pEFαA-*amyM-TR2* | F: CATCCAGgctGCGGAGAGCGGCCTGTACGCGG |
|  | R: TCTCCGCagcCTGGATGCTGACGGTGGATTCA |
| W374A for pEFαA-*amyM-TR2* | F: AGCGGCTCCgctAGCCCCGGCACCGGCTGGAC |
|  | R: GGGCTagcGGAGCCGCTGCCCAGCTTCACCGC |
| P376A for pEFαA-*amyM-TR2* | F: TCCTGGAGCgctGGCACCGGCTGGACGCAGGC |
|  | R: GTGCCagcGCTCCAGGAGCCGCTGCCCAGCTT |
| W380A for pEFαA-*amyM-TR2* | F: CACCGGCgctACGCAGGCCGCCTCCGGCACCG |
|  | R: CCTGCGTagcGCCGGTGCCGGGGCTCCAGGAG |
| W392A for pEFαA-*amyM-TR2* | F: ACCGTGgctACCACCAACACGCCGCCCCCCAC |
|  | R: TTGGTGGTagcCACGGTGTAGTCGGTGCCGGA |

**Table S2 Enzymatic activity of AmyM-TR2 toward various gelatinized and granular starch.**

| The type of gelatinized starch | Enzymatic activity (U/μmol) | | The type of granular starch | Enzymatic activity (U/μmol) | |
| --- | --- | --- | --- | --- | --- |
|  | AmyM | AmyM-TR2 |  | AmyM | AmyM-TR2 |
| PSS | 11600 ± 158 | 5526 ± 18 (0.48) | NWS | 95.2 ± 0.6 | 193.5 ± 3.3 (2.03) |
| NCS | 6497 ± 133 | 4023 ± 26 (0.62) | NCS | 39.0 ± 0.1 | 77.9 ± 2.1 (2.00) |
| CAP | 4890 ± 12 | 4205 ± 72 (0.86) | CAP | 30.8 ± 0.6 | 57.6 ± 0.2 (1.87) |
| CAM | 5291 ± 50 | 2685 ± 30 (0.51) | CAM | 12.5 ± 0.5 | 24.9 ± 0.9 (1.99) |
| CHAM | 8823 ± 116 | 4439 ± 20 (0.50) | CHAM | 18.2 ± 0.5 | 157.7 ± 2.5 (8.66) |

All data are means ± SD (n = 3). Enzymatic activity is expressed as the ratio of specific activity relative to AmyM (values in parentheses).

**Table S3 Binding energies of AmyM and AmyM-TR2 to molecules with varying conformations.**

| Docking molecule-enzyme | | Score (kcal/mol) | ΔΔG (kcal/mol) |
| --- | --- | --- | --- |
| G12 | AmyM | -6.7 | 0.2 |
|  | AmyM-TR2 | -6.5 |  |
| double-helical α-glucan | AmyM | -4.6 | -0.7 |
|  | AmyM-TR2 | -5.3 |  |
| β-cyclodextrin | AmyM | -8.5 | 1.5 |
|  | AmyM-TR2 | -7.0 |  |

**Table S4 Kinetic parameters of AmyM-TR2 toward different type of gelatinized starch.**

| The type of starch | AmyM | | | AmyM-TR2 | | |
| --- | --- | --- | --- | --- | --- | --- |
|  | *K*_m_ (mg/mL) | *k*_cat_ (s^-1^) | *k*_cat_/*K*_m_ (mL·mg^-1^·s^-1^) | *K*_m_ (mg/mL) | *k*_cat_ (s^-1^) | *k*_cat_/*K*_m_ (mL·mg^-1^·s^-1^) |
| PSS | 13.2 ± 0.1 | 24748 ± 64 | 1873 ± 10 | 8.6 ± 0.4 (65.2) | 10882 ± 326 (44.0) | 1264 ± 23 (67.5) |
| NCS | 7.9 ± 0.7 | 11902 ± 561 | 1505 ± 61 | 5.4 ± 0.4 (68.4) | 5760 ± 206 (48.4) | 1067 ± 48 (70.9) |
| CAP | 3.6 ± 0.1 | 6068 ± 40 | 1706 ± 27 | 1.4 ± 0.0 (38.9) | 4718 ± 35 (77.8) | 3438 ± 13 (201.5) |
| CAM | 6.6 ± 0.0 | 8653 ± 1 | 1306 ± 4 | 5.6 ± 0.2 (84.8) | 4265 ± 63 (49.3) | 768 ± 17 (58.8) |
| CHAM | 14.2 ± 0.4 | 20055 ± 179 | 1412 ± 31 | 6.4 ± 0.0 (45.1) | 6499 ± 69 (32.4) | 1014 ± 8 (71.8) |

All data are means ± SD (n = 3). Enzymatic activity and kinetic parameters are expressed as percentages relative to AmyM (values in parentheses).

**Table S5 Docking analysis of AmyM-TR2 and its mutants with double-helix α-glucan molecules.**

|  | H-bond interactions | |
| --- | --- | --- |
|  | Interacting residue | Distance (Å) |
| AmyM-TR2 (WT) | R351 | 3.02(N-O), 3.16(N-O), 2.81(N-O) |
|  | W374 | 3.47(N-O), 3.29(N-O) |
|  | W376 | 3.10(N-O) |
| R351A | K253 | 2.90(O-O), 3.39(O-O) |
|  | Q350 | 2.77(O-O), 2.87(O-O), 3.07(O-O), 3.34(O-O) |
| W374A | A352 | 2.74(N-O), 2.88(N-O) |
|  | S373 | 2.89(O-O), 3.03(O-O) |
|  | G386 | 2.99(O-O), 2.84(O-O) |
|  | W380 | 2.87(O-O), 3.24(O-O) |
| W392A | K330 | 2.78(N-O), 2.86(N-O) |
|  | T381 | 2.67(O-O), 2.88(O-O) |
|  | N395 | 2.85(N-O), 2.99(N-O) |
